# Chronic Antidiabetic Sulfonylureas In Vivo: Reversible Effects on Mouse Pancreatic β-Cells

**DOI:** 10.1371/journal.pmed.0050206

**Published:** 2008-10-28

**Authors:** Maria Sara Remedi, Colin G Nichols

**Affiliations:** Department of Cell Biology and Physiology, Washington University School of Medicine, St. Louis, Missouri, United States of America; Lund University, Sweden

## Abstract

**Background:**

Pancreatic β-cell ATP-sensitive potassium (K_ATP_) channels are critical links between nutrient metabolism and insulin secretion. In humans, reduced or absent β-cell K_ATP_ channel activity resulting from loss-of-function K_ATP_ mutations induces insulin hypersecretion. Mice with reduced K_ATP_ channel activity also demonstrate hyperinsulinism, but mice with complete loss of K_ATP_ channels (K_ATP_ knockout mice) show an unexpected insulin undersecretory phenotype. Therefore we have proposed an “inverse U” hypothesis to explain the response to enhanced excitability, in which excessive hyperexcitability drives β-cells to insulin secretory failure without cell death. Many patients with type 2 diabetes treated with antidiabetic sulfonylureas (which inhibit K_ATP_ activity and thereby enhance insulin secretion) show long-term insulin secretory failure, which we further suggest might reflect a similar progression.

**Methods and Findings:**

To test the above hypotheses, and to mechanistically investigate the consequences of prolonged hyperexcitability in vivo, we used a novel approach of implanting mice with slow-release sulfonylurea (glibenclamide) pellets, to chronically inhibit β-cell K_ATP_ channels. Glibenclamide-implanted wild-type mice became progressively and consistently diabetic, with significantly (*p* < 0.05) reduced insulin secretion in response to glucose. After 1 wk of treatment, these mice were as glucose intolerant as adult K_ATP_ knockout mice, and reduction of secretory capacity in freshly isolated islets from implanted animals was as significant (*p* < 0.05) as those from K_ATP_ knockout animals. However, secretory capacity was fully restored in islets from sulfonylurea-treated mice within hours of drug washout and in vivo within 1 mo after glibenclamide treatment was terminated. Pancreatic immunostaining showed normal islet size and α-/β-cell distribution within the islet, and TUNEL staining showed no evidence of apoptosis.

**Conclusions:**

These results demonstrate that chronic glibenclamide treatment in vivo causes loss of insulin secretory capacity due to β-cell hyperexcitability, but also reveal rapid reversibility of this secretory failure, arguing against β-cell apoptosis or other cell death induced by sulfonylureas. These in vivo studies may help to explain why patients with type 2 diabetes can show long-term secondary failure to secrete insulin in response to sulfonylureas, but experience restoration of insulin secretion after a drug resting period, without permanent damage to β-cells. This finding suggests that novel treatment regimens may succeed in prolonging pharmacological therapies in susceptible individuals.

## Abstract 2

In a mouse study aiming to understand why long-term treatment for type 2 diabetes with sulfonylureas eventually fails, Colin Nichols and Maria Remedi suggest that slow restoration of insulin secretion may be possible after a drug-resting period.

## Editors' Summary

**Background.:**

Diabetes is an increasingly common chronic disease characterized by high blood sugar (glucose) levels. In normal people, blood sugar levels are controlled by the hormone insulin. Insulin is released by β-cells in the pancreas when blood glucose levels rise after eating (glucose is produced by the digestion of food). In fasting people, membrane proteins called ATP-sensitive potassium (K_ATP_) channels keep the β-cell in a “hyperpolarized” state in which they do not secrete insulin. After a meal, glucose enters the β-cell where its chemical breakdown converts ADP into ATP (the molecule that provides the energy that drives cellular processes). The increased ratio of ATP to ADP closes the K_ATP_ channels, “depolarizes” the β-cells, and allows the entry of calcium ions, which trigger insulin release. The released insulin then “instructs” insulin-responsive muscle and fat cells to take up glucose from the bloodstream. In type 2 diabetes, the commonest type of diabetes, the muscle and fat cells gradually become nonresponsive to insulin and consequently blood glucose levels rise. Over time, this hyperglycemia increases the risk of heart attacks, kidney failure, and other life-threatening complications. On average, people with diabetes die 5–10 y younger than people without diabetes.

**Why Was This Study Done?:**

People with type 2 diabetes are often initially treated with drugs called sulfonylureas (for example, glibenclamide). Sulfonylureas help to reduce blood glucose levels by inhibiting (in effect, closing) the K_ATP_ channels, which enhances insulin secretion. Unfortunately, after patients have been treated for several years with sulfonylureas, their β-cells often stop secreting insulin and the patients then have to inject insulin to control their blood sugar levels. The mechanism by which chronic sulfonylurea treatment affects β-cell behavior is poorly understood, which means that it is hard to improve this antidiabetes treatment. Mice that have been genetically altered so that they have no K_ATP_ channels (K_ATP_ knockout mice) also rapidly lose their ability to secrete insulin, although they secrete unusually large amounts at birth. This suggests that permanent membrane depolarization (β-cell hyperexcitability) may cause insulin secretory failure. In this study, the researchers investigate whether this mechanism might be responsible for sulfonylurea-induced loss of insulin secretion.

**What Did the Researchers Do and Find?:**

The researchers implanted slowly releasing pellets of glibenclamide into wild-type mice and then monitored their blood glucose levels and glucose tolerance (the speed of glucose removal from the blood after a glucose “meal”) for up to 128 d; the pellets released drug for 90 d. The glibenclamide-implanted mice progressively developed diabetes, lost the ability to secrete insulin in response to glucose and, after 1 wk of treatment, were as glucose intolerant as adult K_ATP_ knockout mice. Compared to freshly isolated β-cells from untreated wild-type mice, glucose-stimulated insulin secretion by β-cells isolated from glibenclamide-treated wild-type mice and from K_ATP_ knockout mice was reduced to a similar degree. However, the secretory capacity of β-cells isolated from the glibenclamide-treated wild-type mice was restored to normal within hours of drug washout and was normal in β-cells isolated from treated mice 1 mo after exhaustion of the slow-release pellets. Consistent with this result, there was no obvious β-cell death in the glibenclamide-treated mice.

**What Do These Findings Mean?:**

Although findings from animal studies do not always reflect what happens in people, these findings suggest that insulin secretion might sometimes fail in people who take sulfonylureas for a long time, because these drugs cause β-cell hyperexcitability. The finding that the secretory failure caused by sulfonylurea treatment is reversible is important because it suggests that short-acting sulfonylureas might be re-evaluated to see whether they could delay sulfonylurea-induced failure of the insulin secretory response by providing the pancreatic β-cells with periods when they are not depolarized. This finding (and the absence of β-cells death in the glibenclamide-treated mice) also suggests that there may be a way to reverse the loss of the insulin secretory response in patients who have taken sulfonylureas for a long time. Both approaches could help patients with diabetes delay or even avoid the need for insulin injections.

**Additional Information.:**

Please access these Web sites via the online version of this summary at http://dx.doi.org/10.1371/journal.pmed.0050206.

- This study is further discussed in a *PLoS Medicine* Perspective by Renstrom and colleagues
- The MedlinePlus encyclopedia provides information for patients about diabetes (in English and Spanish)
- The US National Diabetes Information Clearinghouse provides information on all aspects of diabetes (in English and Spanish)
- The International Diabetes Federation  also provides comprehensive information about diabetes
- Wikipedia has pages on K_ATP_ channels and on sulfonylurea drugs (note: Wikipedia is a free online encyclopedia that anyone can edit; available in several languages)

## Introduction

Pancreatic β-cell ATP-sensitive potassium (K_ATP_) channels are a critical link between nutrient metabolism and insulin secretion, maintaining the blood sugars in a narrow physiological range. In fasted animals, K_ATP_ channels provide the dominant β-cell membrane conductance, maintaining the cell in a hyperpolarized state and stopping insulin secretion. Conversely, in the fed state, glucose metabolism increases the [ATP]/[ADP] ratio, closing K_ATP_ channels, causing membrane depolarization and voltage-dependent Ca^2+^ entry, which in turn trigger insulin secretion [1].

The K_ATP_ channel is a heteroctameric complex composed of four inwardly rectifying K^+^ channel subunits (Kir6.2) and four sulfonylurea (SU) receptors (SUR1) [2]. Loss-of-function mutations of β-cell K_ATP_ channel subunits (SUR1, *ABCC8*, OMIM accession number 600509; and Kir6.2, *KCNJ11*, OMIM 600937; GenBank, http://www.ncbi.nlm.nih.gov/) underlie congenital hyperinsulinism (HI) in humans [3–7], a genetic disease characterized by relative hyperinsulinemia and hypoglycemia [8]. In HI, reduced or absent K_ATP_ channel activity is expected to result in constitutive depolarization, elevated intracellular [Ca^2+^], and hypersecretion of insulin [9]. In order to replicate the human disease, mice lacking Kir6.2 or SUR1 and mice expressing dominant-negative mutant Kir6.2-encoding transgenes have been generated. Kir6.2- or SUR1-knockout (KO) mice show insulin hypersecretion immediately after birth, but rapidly and unexpectedly progress to glucose intolerance and insulin hyposecretion as adults [10–12]. Conversely, the β-cell–specific Kir6.2[AAA] dominant-negative mouse [13] demonstrates only a ∼70% decrease in β-cell K_ATP_ channel activity, and exhibits insulin hypersecretion with hyperinsulinemia that persist through adulthood. Heterozygous Kir6.2^+/−^ and SUR1^+/−^ mice, which also have reduced K_ATP_ gene dosage (∼60%), also show enhanced glucose tolerance and glucose-sensitive insulin secretion (GSIS) that is maintained throughout adulthood, without progression to secretory failure [14].

We therefore conclude that varying degrees of genetic suppression of K_ATP_ channel activity will all lead to enhanced excitability, but with different long-term consequences for insulin secretion, depending on the severity of suppression: incomplete loss of K_ATP_ channels (e.g., in Kir6.2[AAA] [13] or in heterozygous Kir6.2^+/−^ or SUR1^+/−^ mice [14]) causes a maintained hyperinsulinism, whereas complete loss (in Kir6.2- and SUR1-KO mice) causes transient hypersecretion that is followed by a secretory deficit and reduced glucose tolerance [10–12]. The recombinant phenotypes of many HI mutations [6,15,16] actually suggest that reduced, but not complete, absence of K_ATP_ channels [17] is likely. Henwood et al. [18] have demonstrated that some HI patients with K_ATP_ channel mutations clearly maintain some K_ATP_ channel activity, because the patients were responsive to K_ATP_ channel drugs. There are also limited reports that some HI patients, even those nonsurgically treated, can spontaneously progress to type 2 diabetes [19–21]. HI is also often present at the onset of clinically overt type 2 diabetes, which is interpreted as the endocrine pancreas trying to compensate for primary defects in insulin sensitivity in the peripheral tissues [22]. Thus the progression from relative to absolute insulin deficiency by decreasing insulin secretory capacity appears as the result of a detrimental long-term increased workload of β-cells [23].

SUs, such as tolbutamide and glibenclamide (glyburide), are widely used in patients with type 2 diabetes, because they induce insulin secretion independently of the metabolic state of the β-cell [24–26]. These antidiabetic drugs bind to the SUR1 subunit, leading to inhibition of K_ATP_ channel activity, membrane depolarization, and insulin secretion. Patients with type 2 diabetes chronically treated with SUs often progress to a failure of β-cells to secrete insulin [27–29]. The systemic or cellular mechanism underlying such failure is not well understood, although it could be linked either to the evolution of the disease or to a specific effect of the drugs [30–32]. Consistent with all these studies, the UK Prospective Diabetes Study (UKPDS) revealed that 48% of the patients with type 2 diabetes treated for 6 y with glibenclamide required additional therapy to maintain their normal blood sugars [33].

Despite the widespread use of SUs, there is also evidence that chronic SU (tolbutamide and glibenclamide) treatment may induce Ca^2+^-dependent β-cell apoptosis in rat islets [34] and human islets incubated with glibenclamide demonstrated a significant decrease in insulin content (24 h incubation), as well as an approximate 2-fold increase in β-cell apoptosis (4 d incubation) [35,36]. Glibenclamide-treated Min6 cells reportedly also showed a reversible reduction in insulin content and an accelerated apoptotic β-cell death [37–39], although SU (glibenclamide)-induced apoptosis was apparently specifically enhanced only by expression of the receptor SUR1, but not SUR2B, in HEK 293 cells [40].

The parallels between the long-term consequence of genetic hyperexcitability in mice and desensitization/apoptosis to prolonged SU treatment in humans lead us to hypothesize that, in vivo, SUs will induce an increase in electrical activity leading to enhanced insulin secretion in the short term, but that in the longer term they may still cause membrane hyperexcitability but paradoxically lead to insulin secretory failure, reproducing the K_ATP_ channel KO mouse phenotype. In this paper we specifically tested this hypothesis using a novel pharmacological approach by implanting slow-release (90 d) SU (glibenclamide) pellets.

## Methods

### Animals

Wild-type C57BL/6 mice were obtained from Jackson Laboratories (6-wk-old males, JAX mice; http://jaxmice.jax.org/). Kir6.2 KO mice (a gift from Dr. Susumo Seino) were originally generated by targeted disruption of the gene encoding Kir6.2 in the 129Sv mouse strain [10]. SUR1 KO mice (a gift from Dr. Mark Magnuson) were originally generated in the C57BL/6 mice by pronuclear microinjection of 1 ng/μl *CMV-Cre* expression vector (pBS185) into embryos obtained from a mating of *Sur1^lox+neo^* ^/*w*^ and *Sur1^w^* ^/*w*^ mice [11]. All experiments were performed in compliance with the relevant laws and institutional guidelines, and were approved by the Washington University Animal Studies Committee.

### Acute Glibenclamide Injection in Wild-Type C57BL/6 Mice

Fed wild-type (WT) mice were acutely injected with glibenclamide (0, 1, 3, 10, and 30 μg) and tested for blood glucose levels over time. Intraperitoneal glucose–glibenclamide tolerance tests were performed in fasted (12 h) 6-wk-old WT mice by simultaneous injection of a bolus of glucose (1.5 g/kg) plus glibenclamide at the indicated doses. Glibenclamide responsivity following implantation was assessed by injection of 30 μg/ml in fed mice. Blood was assayed for glucose content using the Glucometer Elite XL (Bayer, http://www.bayer.com).

### Blood Glucose and Plasma Insulin Measurements on Mice Implanted with Glibenclamide Pellets

Glibenclamide (glyburide) pellets at the concentration of 0.0001, 0.001, 0.025, 0.25, and 2.5 mg per 90-d release were obtained from Innovative Research of America (Sarasota, FL). Six-week old males were anesthetized with tribromoethanol (Avertin; 0.25 mg/g mouse body weight). The skin on the lateral side of the neck of the animal was lifted and pellets were implanted under the skin of the neck using a stainless steel precision trochar. Blood samples taken from the tail vein in fed and fasting conditions, and during glucose tolerance tests (GTTs), were assayed for glucose content as described above. Intraperitoneal GTTs were performed in WT, Kir6.2-KO, and SUR1-KO mice implanted with different glibenclamide pellet concentrations. For insulin tolerance tests (ITTs), mice were injected with 0.5 U insulin/kg following 6-h fasting. Blood was taken at different times (as indicated in figures) and assayed for glucose content. Plasma insulin was measured on glibenclamide-treated mice at 2 d and 42 d after pellet implantation using a rat insulin ELISA kit.

### Islet Isolation

Mice were anesthetized with halothane (0.2 ml) in an anesthetizing chamber and killed by cervical dislocation. Pancreata were removed and injected with Hank's balanced salt solution (HBSS; Sigma-Aldrich, http://www.sigmaaldrich.com/) containing collagenase (0.3 mg/ml) (pH 7.4). Collagenase type XI was obtained from Sigma-Aldrich. Pancreata were digested for 5 min at 37 °C, hand-shaken, and washed three times in cold HBSS solution [41]. Islets were isolated by hand under a dissecting microscope and pooled islets were maintained in CMRL-1066 culture medium (GIBCO) supplemented with fetal calf serum (FCS, 10%), penicillin (100 units/ml), and streptomycin (100 μg/ml).

### ^86^Rb^+^ Efflux Experiments

Isolated islets were pre-incubated with ^86^Rb^+^ (rubidium chloride 1.5 mCi/ml, Amersham Biosciences) for 1 h. Loaded islets were placed in microcentrifuge tubes (30 per group) and washed twice with RPMI-1640 media (Sigma-Aldrich). ^86^Rb^+^ efflux was assayed by replacing the bathing solution with Ringer's solution with metabolic inhibitor (MI), with or without 1 μmol/l glibenclamide. MI solution contained 2.5 mg/ml oligomycin, 1 mM 2-deoxyglucose, together with 10 mM tetraethylammonium to block voltage-gated K^+^ channels, 10 μM nifedipine to block Ca^2+^ entry, and 30 mM KCl to maintain Em ∼ 0. The bathing solution was replaced with fresh solution every 5 min over a 40 min period, and counted in a scintillation counter. ^86^Rb efflux was fitted by a single exponential and the reciprocal of the exponential time constant (rate constant) for each efflux experiment is then proportional to the K^+^ (Rb^+^) conductance of the islet membranes.

### Insulin Release Experiments

Acutely, following isolation, or after overnight incubation in low-glucose (5.6 mM) CMRL-1066, islets (ten per well in 12-well plates) were preincubated for 30 min in glucose-free CMRL-1066 plus 3 mM D(+)-glucose, then incubated in CMRL-1066 plus different glucose concentration, as indicated. Islets were incubated for 60 min at 37 °C and medium removed and assayed for insulin content. Isolated islets were sonicated on ice prior to estimation of insulin content. Insulin was measured using rat insulin radioimmunoassay according to manufacturer's procedure (Linco, http://www.millipore.com). Experiments were repeated in triplicate.

### Immunohistological Analysis

Pancreata from WT, Kir6.2 KO, and SUR1 KO mice treated for 56 d with placebo or glibenclamide pellets were fixed in 10% formalin and paraffin embedded for serial sectioning (5 μm thick). Hematoxylin-eosin (HE) staining was carried out as described previously [42]. For insulin and glucagon immunofluorescence, pancreatic sections were incubated overnight at 37 °C with a guinea pig anti-insulin primary antibody or a guinea pig anti-glucagon primary antibody (1:250 or 1:500, respectively; Linco). Primary antibodies were detected by incubating for 1.5 h at 25 °C with an anti-guinea pig secondary antibody conjugated with the Alexa 488 fluorescent dye (Molecular Probes, http://www.invitrogen.com) [42]. Apoptosis was assessed on paraffin sections using the TUNEL staining technique (ApopTag Plus Fluorescein In Situ Apoptosis Detection Kit, Chemicon International, httpp://www.millipore.com). DNAase I recombinant (Roche) was used to damage DNA as a positive control for the experiment.

### Statistics

Data are presented as mean ± standard error of the mean (SEM). Differences between the non-implanted control group and the pellet-implanted groups were assessed using analysis of variance (ANOVA) within each time point. When significant and within the framework of the ANOVA, the Duncan post hoc test was used to test specific hypotheses about the equivalence of the control group with each test group. When only two groups were compared, unpaired *t*-tests were used to assess significance. Differences were assumed to be significant in each case if *p* < 0.05. When the sample size was three or less, statistics were not performed and significance is not assigned. Given sample size limitations, we did not use a multilevel model to compare change over time between groups. Unless stated otherwise, asterisks in figures indicate significant difference between the test group and the control group within time point or condition, non-significant differences are not indicated.

## Results

### Glucose Tolerance Is Enhanced and Fed Glucose Is Reduced in WT Mice Acutely Injected with Glibenclamide

**Figure 1:**
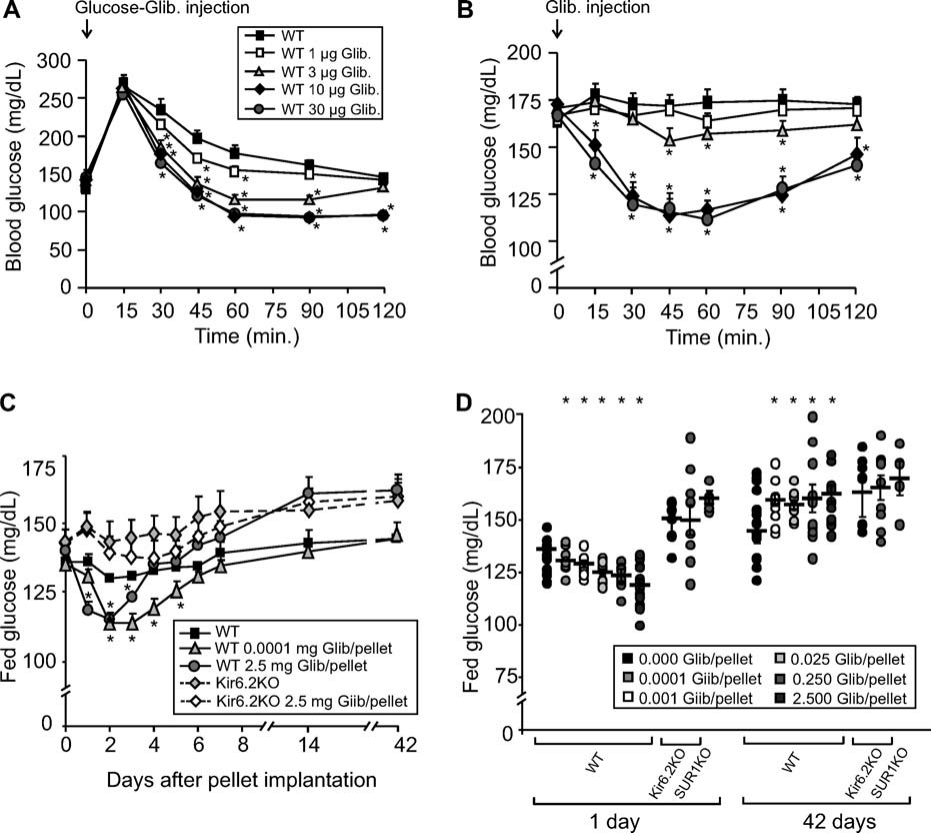
Acute and Chronic Effect of Glibenclamide on Glucose Tolerance and Blood Glucose Levels In each graph, asterisks indicate control group is significantly different (*p* < 0.05) from the test group at the specified time point. (A) GTTs on 6-wk-old WT mice (*n* = 10) fasted for 12 h and intraperitoneally injected with 1.5 g/kg glucose and glibenclamide simultaneously. (B) Blood glucose response of fed 6-wk-old mice (*n* = 10) to acute injection of glibenclamide. (C) Fed blood glucose from WT and Kir6.2 knockout (Kir6.2KO) mice implanted with placebo or glibenclamide pellets (0.0001 or 2.5 mg/pellet) over time. (D) Individual values of fed glucose (plus mean and SEM) from WT control and glibenclamide-pelleted mice, as well as Kir6.2-KO and SUR1-KO mice implanted with 2.5 mg pellets, at 1 and 42 d after implantation. Each group in (C) and (D) contained ten to 15 mice.

Six-week-old C57BL/6 WT mice simultaneously injected with 1.5 g/kg glucose and glibenclamide (0, 1, 3, 10, and 30 μg) showed a dose-dependent enhancement of glucose tolerance, with a more rapid glucose decline compared with control mice injected with only glucose (Figure 1A). Consistent with the well-known acute effect of SUs on blood sugars, and in correlation with the glibenclamide effect on the GTT, these acute injections caused a rapid, dose-dependent drop in blood glucose levels. Importantly, the two highest concentrations of glibenclamide (10 and 30 μg) caused a similar marked reduction of fed blood glucose after an extended period, consistent with a saturated effect of the drug in vivo (Figure 1B).

### Biphasic Blood Glucose Response of WT Mice Chronically Treated with Glibenclamide

As discussed in the Introduction, K_ATP_ KO mice show an unexpected phenotype that contradicts the simple prediction for complete inhibition of K_ATP_ channel activity [10–12]. We have previously shown that this phenotype depends on complete, or nearly complete loss of K_ATP_, while intermediate loss induces insulin hypersecretion [14]. We suggest that this biphasic response profile is a direct consequence of loss of K_ATP_ and resultant hyperexcitability. In order to pharmacologically test this hypothesis, we took advantage of the availability of slow-release implantable drug pellets. Mice were chronically treated with glibenclamide, a specific K_ATP_ channel blocker, by pellet implantation at 6 wk of age. We could thus examine long-term (90 d) effects of the drug, avoiding the peak and valley effect produced by single injections and the stress of frequent injections.

During the first four days following implantation with high-dose slow-release glibenclamide pellets, fed blood glucose levels in WT mice were significantly lower than in placebo-implanted mice (Figure 1C), and the glucose-lowering effect was glibenclamide-dose–dependent. The circulating glibenclamide level is unknown in these experiments. However, therapeutic glibenclamide doses in humans are typically around 0.05 mg/kg/d. Pellets containing 0.1 mg released over 90 d should give a similar net dose in a 25 g mouse; thus, the implanted doses (0.0001–2.5 mg) should span therapeutic ranges. The initial decrease in fed blood sugars was lost within a few days, and fed blood glucose was significantly higher in implanted animals after 4–5 d, for all implanted doses except the very lowest 0.0001 mg dose (Figure 1C). Two weeks after pellet implantation, all glibenclamide-treated mice (with the exception of those with the 0.0001 mg pellet) showed similar elevated fed blood glucose, and this elevated level persisted without further impairment until 42 d after implantation (Figure 1C). The elevation was essentially the same as that seen in placebo-treated K_ATP_ KO mice, with no significant differences between glibenclamide- and placebo-treated KO mice (Figure 1C and 1D).

### Impaired Glucose Tolerance in WT Mice Chronically Treated with High Doses of Glibenclamide

**Figure 2:**
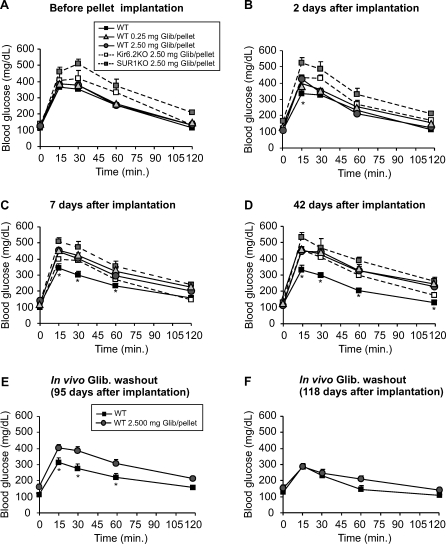
Impaired Glucose Tolerance in Mice Implanted with High Dose Slow-Release Glibenclamide Pellets GTTs on WT mice before (A), 2 d (B), 7 d (C), 42 d (D), 95 d (E), and 118 d (F) after implantation of pellets containing high doses of glibenclamide. Each group contained ten mice, except five in (E) and (F). Mice were injected intraperitoneally with glucose (1.5 g/kg). Blood was taken at times indicated and assayed for glucose concentration. In each graph, asterisks indicate control group is significantly different (*p* < 0.05) from the test group at the specified time point.

As previously reported, Kir6.2 KO and SUR1 KO mice are less glucose tolerant than WT mice [10–12], as illustrated by intraperitoneal GTT (Figure 2A). WT mice implanted with high-dose glibenclamide pellets (0.25 and 2.5 mg/pellet, equivalent to a release of 3 and 30 μg/d) showed very rapid progression to impaired glucose tolerance compared to WT placebo-treated mice (Figure 2B–2D). After only 7 d (Figure 2C), and sustained thereafter to at least 42 d postimplantation (Figure 2D), glibenclamide-treated mice were as glucose intolerant as KO mice. Moreover, K_ATP_ KO mice chronically treated with glibenclamide show no change in the preexisting glucose-intolerant response. Thus, pharmacological block of the K_ATP_ channel in WT mice essentially reiterates the loss of glucose tolerance seen in K_ATP_ channel KO mice.

### Enhanced Glucose Tolerance, but Crossover to a Progressive Impairment of Glucose Tolerance, in Mice Implanted with Low-Dose Glibenclamide Pellets

**Figure 3:**
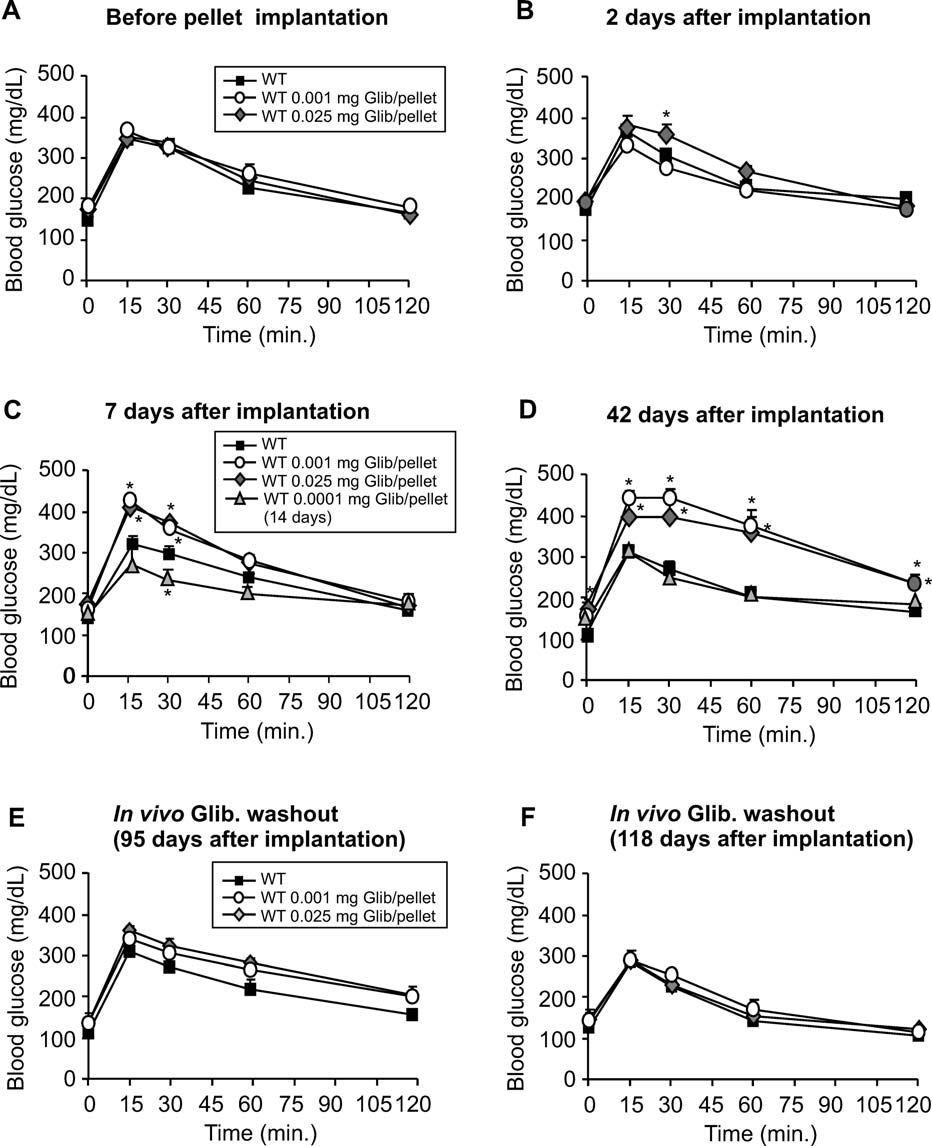
Enhancement and Crossover of Glucose Tolerance in Mice Implanted with Low Dose Slow-Release Glibenclamide Pellets In each panel, asterisks indicate control group is significantly different (*p* < 0.05) from the test group at the specified time point. GTTs performed on WT mice before (A), 2 d (B), 7 d (C), 42 d (D), 95 d (E), and 118 d (F) after implantation of pellets containing high doses of glibenclamide. Groups contained ten mice each, except five in (E) and (F). Note: The lowest-dose pellets (0.0001 mg) were examined only at 14 and 42 d postimplant.

GTTs were also performed in mice implanted with pellets containing much lower doses of glibenclamide (Figure 3). An early (2 or 7 d after implantation) mild enhancement of glucose tolerance was detectable in mice implanted with a very low dose of glibenclamide (0.001 or 0.0001 mg/pellet, Figure 3B and 3C, respectively), but this was again followed by a progressive impairment in glucose tolerance over time (Figures 3C and 3D). Again, 42 d postimplantation, 0.001 mg/pellet–treated mice showed similar impairment in glucose tolerance to mice treated with high doses of glibenclamide for the same period of time (Figures 2D and 3D). However, this crossover was markedly delayed with the very lowest dose (0.0001 mg/pellet); at 7 d glucose tolerance was significantly enhanced, although at 42 d, blood glucose and glucose tolerance were similar to control. Thus, at very low doses, the “pelleted” mice resemble heterozygous K_ATP_ knockout mice [13,14,43], at least for a period of 2–7 d.

### In Vivo Dose- and Time-Dependent Changes in Insulin Secretion in Response to Glucose Injection in Mice Chronically Treated with Glibenclamide Pellets

**Figure 4:**
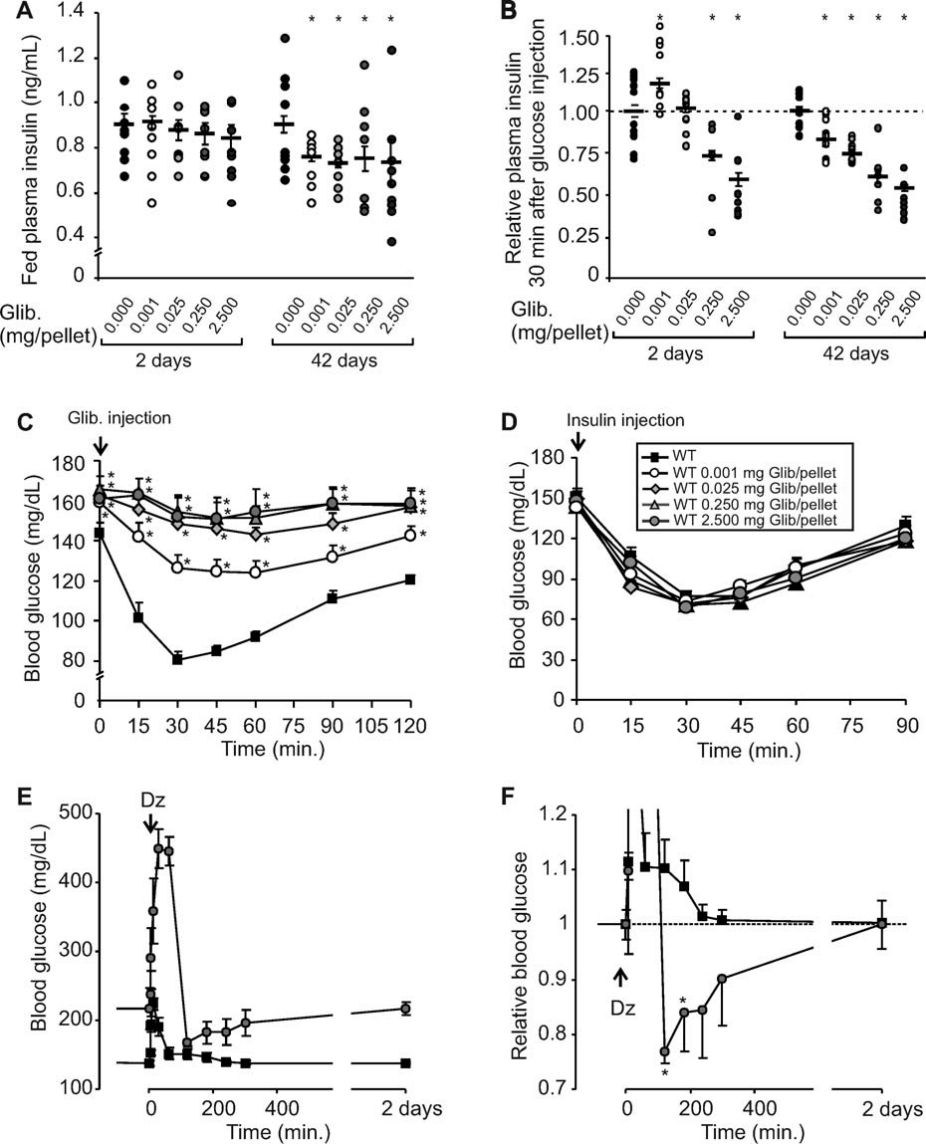
Drug and Insulin Responsivity In Vivo In each graph, asterisks indicate control group is significantly different (*p* < 0.05) from the test group at the specified time point. (A) Individual fed plasma insulin (plus mean and SEM) in glibenclamide-treated mice, 2 d and 42 d after glibenclamide implantation. (B) Individual values (plus mean and SEM) of plasma insulin 30 min after glucose injection (normalized to control unpelleted) at either 2 d or 42 d. (C and D) WT mice 5 wk implanted with different doses of glibenclamide were intraperitoneally injected with glibenclamide (C) or with insulin (D) and blood glucose was assessed over time. (E and F) Control and high-dose glibenclamide-pelleted mice were intraperitoneally injected with 20 mg/kg diazoxide, and blood glucose was assessed over time. In (F), glucose is normalized to the precontrol value (indicated by broken line).

Two days after implantation, plasma insulin was very slightly elevated in mice treated with 0.001 mg pellets, but was dose-dependently reduced in all mice treated with higher doses for the same period. At 42 d after glibenclamide treatment, fed plasma insulin was significantly reduced in all implanted mice (Figure 4A), paralleling the marked increase in blood sugar (Figure 1D), independent of the glibenclamide dose. Glucose-stimulated insulin secretion (GSIS) in vivo was assessed from plasma insulin values 30 min after glucose challenge. Insulin secretion in response to glucose was increased in mice treated with the lowest dose of glibenclamide, consistent with the enhanced glucose tolerance observed in these mice 2 d after drug implantation. Also consistent with changes in glucose tolerance, all other glibenclamide-implanted mice showed a dose-dependent reduction in insulin secretion that worsened over time (Figure 4B).

### No Effect of Acute Glibenclamide Injection, and Normal Insulin Sensitivity in Mice Chronically Treated with Glibenclamide

To test whether the glucose-intolerant phenotype of chronically glibenclamide-treated mice is still correlated with loss of function of K_ATP_ channel activity, the response to acute injection of glibenclamide was measured at the times indicated. As expected for complete or near complete inhibition of the channel by the circulating drug levels, injection of glibenclamide produced no significant effect in mice chronically treated with high-dose pellets, and only a modest lowering of blood sugars in mice implanted with low-dose pellets (Figure 4C).

To assess insulin sensitivity in peripheral tissues, WT placebo- and glibenclamide-implanted mice were subjected to an ITT. Mice implanted with both low and high doses of glibenclamide showed similar insulin sensitivity to WT-placebo-implanted mice (Figure 4D).

### Glibenclamide-Treated Islets Exhibit a Marked Reduction of K_ATP_ Conductance

**Figure 5:**
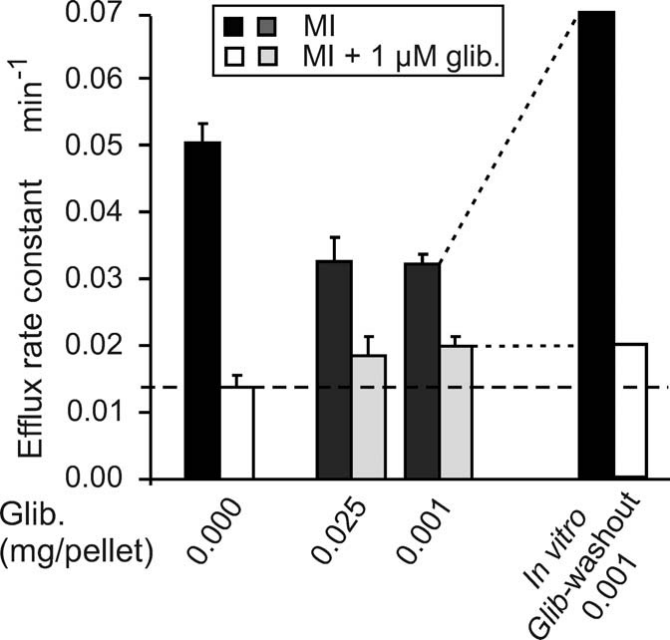
Glibenclamide-Treated Islets Exhibit a Reduction of Total K_ATP_ Channel Density Macroscopic K_ATP_ channel density in intact islets from WT placebo- and glibenclamide-treated mice was assessed by ^86^Rb^+^ effluxes under metabolic inhibition (MI) in the presence and absence of glibenclamide (glib.). Fluxes were fitted by single exponentials and reciprocal rate constant plotted in the figure (each case contained three animals; no significance assessed). Broken line indicates K_ATP_-independent rate constant, determined by glibenclamide in control islets.

Macroscopic K_ATP_ channel density in freshly isolated (within 2 h) intact islets was assessed by ^86^Rb^+^ efflux under metabolic inhibition to lower cellular [ATP]:[ADP] and maximally activate available K_ATP_ channels (Figure 5). The efflux rate constant (proportional to available K_ATP_ conductance) was significantly reduced in glibenclamide-implanted mice compared with placebo-treated mice, indicating reduced K^+^ conductance. In WT placebo-treated islets, over 70% of the flux was glibenclamide-sensitive; however, WT glibenclamide-treated islets showed less than 40% glibenclamide-sensitive effluxes (Figure 5), indicating that K_ATP_ conductance was substantially reduced. Consistent with a reversible effect of the drug to inhibit the channel directly, maintenance of isolated islets from glibenclamide-implanted mice in the absence of the drug for 24 h (in vitro drug washout) show a dramatic recovery of glibenclamide-sensitive K^+^ conductance, without changes in glibenclamide-insensitive ^86^Rb^+^ effluxes (Figure 5).

The assay of freshly isolated islets was made within 2 h of isolation, at which point there is clearly significant inhibition of K_ATP_ conductance in islets from implanted mice. However, realizing that full recovery occurs within 24 h, it seems likely that some recovery occurs even within this 2 h, so the measured K_ATP_ activity at that time is at best an upper limit estimate, and channel activity in vivo may have been completely suppressed by the circulating glibenclamide levels.

### Chronically Glibenclamide-Treated Mice Show Reduced GSIS from Fresh Isolated Islets, but Restored Normal Insulin Secretion in Drug-Washout Islets

**Figure 6:**
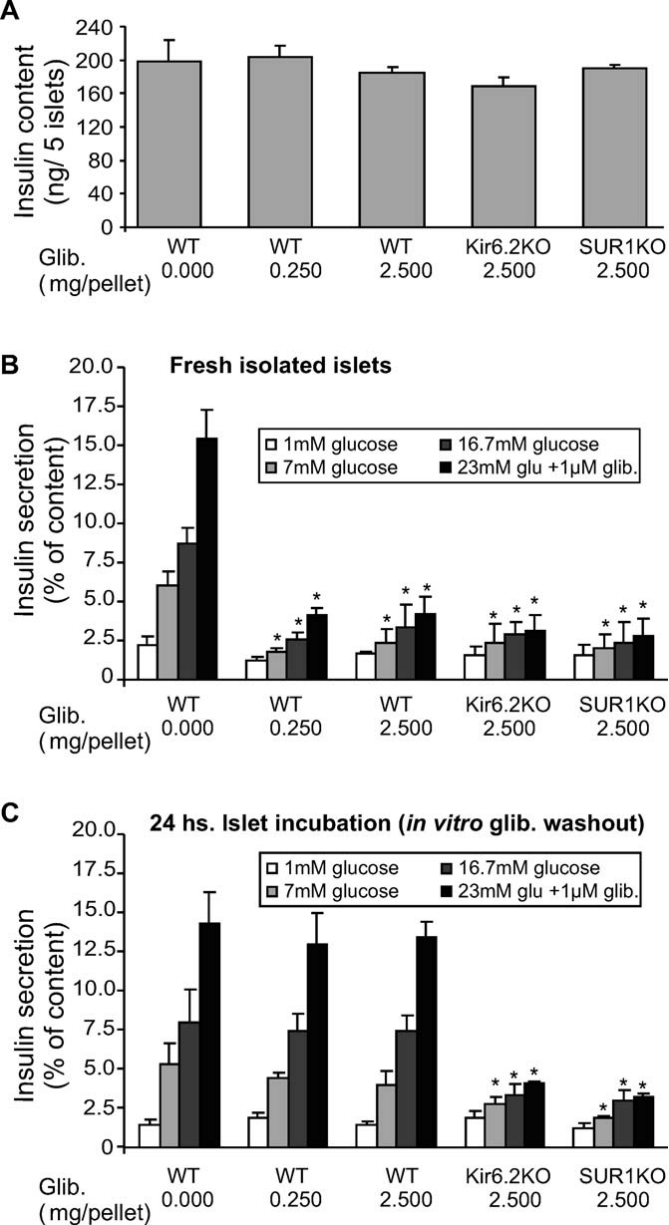
Impaired Glucose-Dependence of Insulin Secretion in Fresh Isolated, but Not in 24-h Incubated Islets from Mice Chronically Treated with Glibenclamide (A) Insulin content from glibenclamide-pelleted mice. (B and C) Glucose-sensitive insulin secretion from freshly isolated islets (B) and from islets incubated in 5.6 mM glucose for 24 h (C). Insulin release and content was measured by radioimmunoassay. Each group contained ten mice, and samples were assayed in triplicate. Significant differences between glibenclamide-implanted and control are indicated for each assay condition by asterisk. There were no significant differences in (A).

In order to assess the effect of long-term glibenclamide treatment on β-cell function directly, pancreatic islets were isolated from either WT or K_ATP_ KO mice after treatment with glibenclamide for 42 d. Insulin content of KO islets was slightly lower than WT islets, but insulin content was not significantly different between placebo- and pellet-implanted animals (Figure 6A). However, while freshly isolated islets from WT placebo-treated mice showed robust glucose- and glibenclamide-dependent insulin secretion, glibenclamide-treated WT and K_ATP_ KO islets both showed a similar, severely blunted response to both glucose and glibenclamide (Figure 6B). A 24 h incubation in CMRL medium (5.6 mM glucose, glibenclamide washout) restored normal glucose and glibenclamide responses in islets obtained from WT glibenclamide-treated mice (Figure 6C), paralleling the recovery of K_ATP_ conductance. However, as expected, insulin secretion was not improved in islets from glibenclamide-treated K_ATP_ KO mice.

### Reversal of Phenotype Is also Observed In Vivo

The above results dramatically demonstrate rapid reversibility in vitro of the chronic glucose-desensitizing effects of glibenclamide. Removal of pellets to assess reversibility in vivo is impractical, so instead we examined glucose control in implanted mice, beyond the time at which release should cease (95 or 118 d). In this case, the animals should have been exposed to glibenclamide for the full 90-d release period, but then should have had either 5 d or 28 d without drug exposure. Partial restoration was detectable at 5 d after drug release (Figures 2E and 3E) was terminated, and even mice that had been implanted with 2.5 mg glibenclamide/pellet showed dramatic restoration of both insulin secretion and glucose tolerance 118 d after pellet implantation (i.e., 28 d after the drug release was terminated) (Figures 2F and 3F).

The restoration of secretion following the cessation of glibenclamide release raises the question of whether secretion might also be acutely restored by diazoxide treatment (to activate K_ATP_ and block the hyperexcitability) during the period of glibenclamide release. Acutely, diazoxide may cause glucose to rise, but in “resting” the β-cells may also transiently allow recovery of insulin “releasability,” leading to a transient restoration of normal glucose. We performed the experiment on control and high-dose (2.5 mg) pelleted animals. As predicted, there was a rise in glucose in both cases, but a marked subsequent prolonged undershoot only in the pelleted animals (Figure 4E and 4F).

### Morphology of Pancreas and α-/β-Cell Distribution within Islets from Glibenclamide-Implanted Mice Is Not Altered

**Figure 7:**
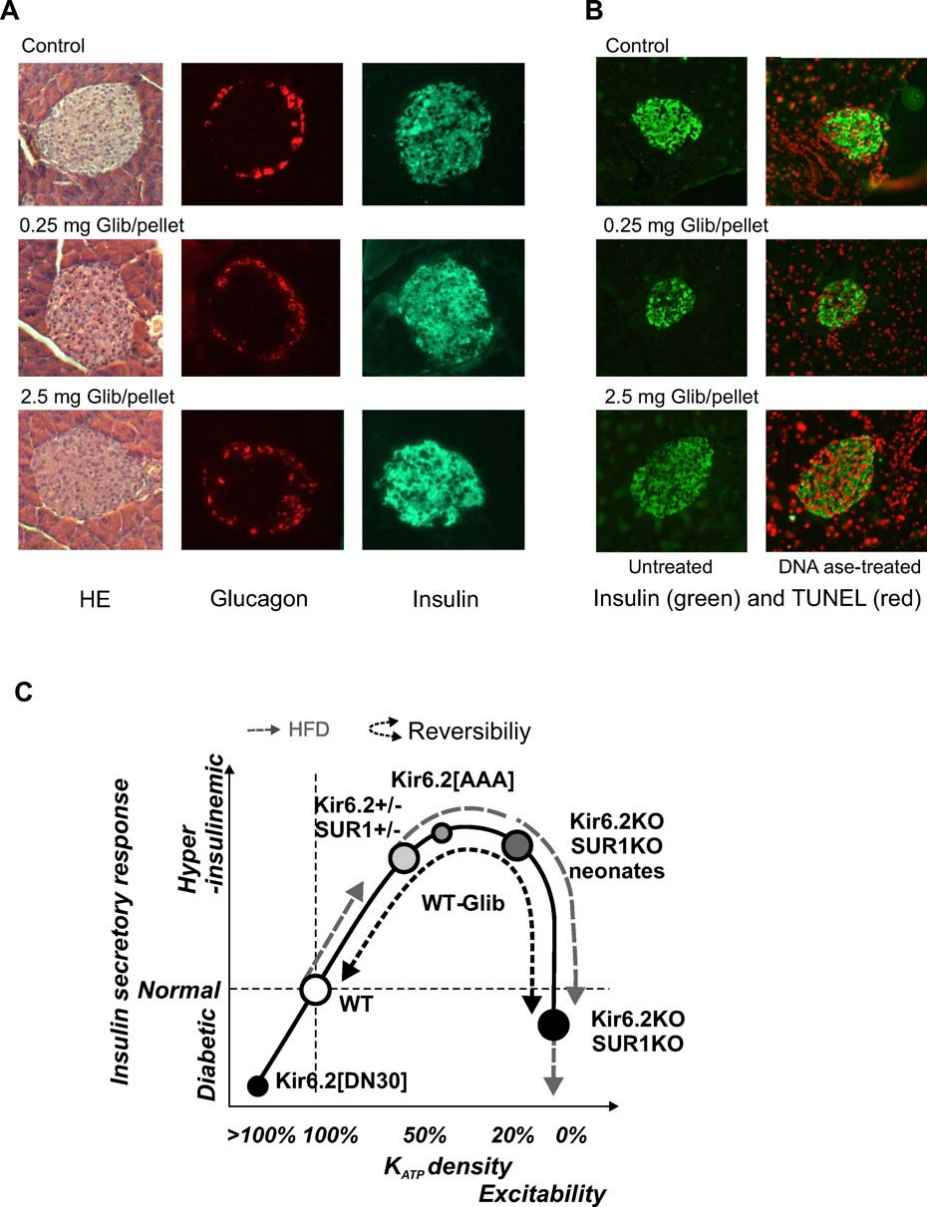
Morphological and Physiological Response to Chronic Hyperexcitability, and Proposed “Inverse U” Model Response for Enhanced β-Cell Excitability (A) Hematoxylin-eosin staining (left), glucagon (middle), and insulin (right) immunostaining of pancreatic paraffin sections from WT control and glibenclamide-implanted mice. (B) Insulin (green) and TUNEL (red) staining of pancreatic sections from control and glibenclamide-pelleted mice. Left images show images from untreated sections; right images show islets from paraffin sections treated with recombinant DNAase I (TUNEL-positive controls). (C) Proposed “Inverse U” model response for enhanced β-cell excitability: normal islets (white circle) secrete normally, but following a high-fat diet (HFD; grey dashed arrow) progress to insulin hypersecretion. Both Kir6.2[AAA] and heterozygous Kir6.2- and SUR1-KO mice (50%–70% decreased K_ATP_ activity with increased excitability) hypersecrete (grey circles, solid line) and are positioned on the “ascending limb” of the curve [13,14,43]. HFD causes further enhancement of excitability, beyond the threshold driving those islets “over the top” (dashed) to an undersecretory phenotype. Conversely, Kir6.2- and SUR1-KO islets (zero K_ATP_ channel activity), which have maximally enhanced excitability, hypersecrete as neonates (grey circle on solid line), but rapidly progress to an undersecretory phenotype (black circle on solid line), and are positioned on the “descending limb” [10–12]. Glibenclamide-treated WT mice rapidly progress from normal insulin secretion to undersecretion (white circle on “ascending limb” is converted to black circle on “descending limb”). This phenotype can be completely reversed when hyperexcitability is removed (small black dashed line). Finally, mice expressing mutant β-cell K_ATP_ channels with enhanced activity (Kir6.2[ΔN30]) [41] have a severely undersecreting phenotype, extending the ascending limb beyond the position of normal animals.

The normal content of insulin and rapid recovery of secretion in isolated islets from glibenclamide-treated animals would seem to rule out apoptotic or necrotic effects of the drug on β-cells. However, to confirm this, immunohistochemical analyses were performed on sectioned pancreata from 9-wk glibenclamide-implanted mice. Hematoxylin-eosin staining showed similar pancreatic architecture in glibenclamide-implanted vs. placebo-treated mice (Figure 7A, left photomicrographs). Immunostaining for insulin and glucagon confirmed normal distribution of both insulin-containing β-cells and glucagon-containing α-cells, with no obvious changes in islet size (Figure 7A, middle and right photomicrographs). No marked redistribution of α-/β-cells or loss of β-cell mass (i.e., reduced insulin immunofluorescence) was observed in glibenclamide-treated mice, although there was a very slight tendency for α-cell infiltration in the core of glibenclamide-treated pancreatic islets. In order to specifically test for the possibility of enhanced β-cell death, in situ immunostaining for apoptosis was performed in paraffin-fixed sections using the TUNEL technique (Figure 7B). Pancreatic sections were costained with insulin (green) and TUNEL (red). The absence of red staining in the left images of Figure 7B indicates no obvious β-cell apoptosis was present in islets from control or high dose (0.25 and 2.5 mg/pellet) glibenclamide-pelleted mice. As positive control, consecutive pancreatic sections from each sample were treated with recombinant DNAase I, resulting in extensive TUNEL positivity in endocrine as well as exocrine cells (Figure 7B, right photomicrographs).

## Discussion

In the present study, we examined the “inverse U” hypothesis to explain the response to enhanced excitability, in which excessive hyperexcitability drives β-cells to insulin secretory failure [14,42], using a novel approach of implanting mice with slow-release sulfonylurea (glibenclamide) pellets, to chronically inhibit β-cell K_ATP_ channels. Glibenclamide-implanted wild-type mice became progressively and consistently diabetic, with significantly reduced insulin secretion in response to glucose. After 1 wk of treatment, these mice were as glucose intolerant as adult K_ATP_ knockout mice, with similar loss of secretory capacity. However, secretory capacity was fully restored in these islets within hours of drug washout in vitro, or within 1 mo after glibenclamide treatment was terminated in vivo. Pancreatic immunostaining showed normal islet size and α-/β-cell distribution within the islet, and TUNEL staining showed no evidence of apoptosis.

### Hyperexcitability and Hyperinsulinism in Animal Models and Humans: Key Features and Discrepancies

Glucose metabolism increases the β-cell [ATP]:[ADP] ratio, leading to closure of K_ATP_ channels, membrane depolarization and Ca^2+^ influx. The resultant rise in intracellular Ca^2+^ ([Ca^2+^]_i_) triggers insulin secretion. Reduced or absent K_ATP_ channel activity is expected to result in constitutive membrane depolarization, elevated [Ca^2+^]_i_, and hypersecretion of insulin. Consistent with this prediction, loss-of-function mutations of K_ATP_ channel subunits (SUR1, *ABCC8* or Kir6.2, *KCNJ11*) underlie congenital HI in humans [9]. Conversely, gain-of-function mutations of Kir6.2 or SUR1 are expected to cause an undersecreting phenotype and hypoinsulinemia—and consistent with this prediction, activating mutations in K_ATP_ cause both permanent and transient neonatal diabetes [44,45].

Genetic manipulation of K_ATP_ channel subunits in mice has confirmed some of the basic expectations of the above paradigm and reiterated some features of human disease, although conflicting and contradictory findings illustrate additional complexities, particularly for models of reduced K_ATP_ channel activity. Mice that completely lack K_ATP_ channels do not reiterate the HI phenotype in any simple way. In these mice, hypersecretion reportedly occurs immediately after birth, but there is rapid progression to a relative undersecretion and glucose intolerance [10–12]. There is thus an apparent discrepancy between the outcome of genetic loss of K_ATP_ channels in mice and humans. One important potential caveat to consider is whether patients typically have a complete loss of K_ATP_ channels, and therefore whether complete KO mice are appropriate models for the disease. Both trafficking and functional mutations will probably tend to cause only a relative loss of K_ATP_ conductance. Mice expressing a dominant-negative Kir6.2[AAA] transgene in β-cells, as well as mice heterozygous for Kir6.2 or SUR1, may model such a condition, since they show substantial, but incomplete, reduction of K_ATP_ channel activity (∼60%–70%). All of these models demonstrate both an enhanced glucose stimulation of insulin secretion and hyperinsulinism that persists through adulthood [13,14,43].

### Biphasic Response to K_ATP_ Inhibition: Pharmacological Verification of the “Inverse-U” Model for β-Cell Response to Hyperexcitability

We therefore propose that varying degrees of genetic suppression of K_ATP_ channels will all lead to enhanced excitability and insulin secretion, but with potentially very different long-term consequences depending on the severity of suppression: incomplete loss of K_ATP_ channels (e.g., in Kir6.2[AAA] or in heterozygous Kir6.2^+/−^ or SUR1^+/−^ mice) causes a persistent hyperinsulinism, whereas complete loss (e.g., in K_ATP_ KO mice) may transiently cause hypersecretion, but this is followed by a secretory deficit and reduced glucose tolerance (Figure 7C) [14,42].

We suggest that this model may be generalizable to hyperexcitability resulting from any other mechanism, such as loss of other K^+^ conductances, or gain of excitatory current. However, it is important to note that the model is developed solely from experiments with genetic manipulation of K_ATP_ channel subunits, and requires verification by direct manipulation of excitability in genetically normal animals. In the present study we have successfully performed just such an experiment by utilizing slow-release implanted drug pellets. This approach allowed us to pharmacologically block K_ATP_ channels over a prolonged period in a tractable in vivo model. The dramatic consequence is that although blood glucose levels are initially lowered and glucose tolerance is enhanced (Figures 1 and 3), within a few days the phenotype crosses over to hyperglycemia and glucose intolerance that is strikingly similar to the adult phenotype of K_ATP_ KO mice for all except the very lowest-dose pellets. Earlier studies of “long-term” treatment of animals with sulfonylureas have generally not gone beyond a few days [46], presumably due to the practical difficulties of injection regimens. Importantly, young mice injected daily with glibenclamide showed a degranulation effect in their β-cells, which might explain the reduced secretory capacity, although this effect was apparently not present in older mice [47]. Increased basal insulin secretion but reduced glucose- and glibenclamide-stimulated insulin secretion have been seen from isolated islets exposed in vitro to glibenclamide for 24 h [48,49], due to prolonged glibenclamide action and reduced insulin content [49]. Similarly, chronic exposure of rat pancreatic islets to sulfonylureas caused reversible impairment of glucose- and sulfonylurea-induced insulin release [50–52]. While short-term (24 h) exposure of insulinoma cell lines to SUs induced an increase in GLUT2 and GK mRNA, long-term (48–72 h) exposure induced a marked reduction of these glucose mobilizers [53], coupled with a reversible reduction in insulin content with no changes in Kir6.2 or SUR1 transcripts (72–144 h) [39]. Here we demonstrate that intact mice treated with chronic glibenclamide show no significant changes in islet insulin content, although a very mild reduction in insulin content was found in islets from mice implanted with the highest dose (Figure 6). Instead, our results indicate very specifically that chronic glibenclamide treatmen*t* in vivo induces β-cell desensitization (impairment of insulin secretion) rather than inhibition of insulin production [54]. ^86^Rb^+^-efflux experiments on fresh isolated islets from glibenclamide-implanted mice demonstrate that substantial inhibition of K_ATP_ activity is present (Figure 5), which predicts hyperexcitability and elevated [Ca^2+^], as has been seen in K_ATP_ KO animals. This would in turn stimulate insulin secretion, which suggests that the impairment of secretion is at a stage downstream of Ca^2+^, presumably at the level of insulin production or mobilization, or at the level of β-cell mass. There is a remarkable and rapid recovery of both K_ATP_ activity and normal insulin secretion within 24 h of islet isolation from these mice, arguing against β-cell apoptosis or other types of cell death induced by chronic glibenclamide treatment in vivo, and supported by the immunhistological analyses in Figure 7A and 7B.

Clearly, recovery of both K_ATP_ conductance and secretion is rapid (within hours of removal of islets from the animal), and although unassessable, the most reasonable explanation is that the recovery results simply from the washout of the drug and reversal of the resultant chronic depolarization. However, the formal possibility remains that the recovery is due to loss of other neuronal or hormonal inputs present in the intact animal. The level of K_ATP_ activity in Figure 5, assessed about 2 h after isolation, is an upper limit estimate, since some recovery may already have occurred and, in vivo, the degree of inhibition may be considerably greater. That this is likely is illustrated in Figure 5: except for the very lowest dose of implanted glibenclamide pellets, the glucose-lowering effect of injected glibenclamide, which reflects the degree of K_ATP_ inhibition possible, was negligible.

### The “Inverse U” Model Is Reversible: Removal of the Hyperexcitable Stimulus Restores Normal Responsivity

In the genetic models of hyperexcitability, progression onto the “descending limb” of the “inverse U” response is permanent; glucose intolerance persists throughout the lifetime of K_ATP_ KO animals, and in reduced K_ATP_ animals when under dietary stress. However, it appears that this progression is rapidly reversible if excitability is normalized (as we show here), or if the dietary stress is removed [42]. As we discuss further below, various studies have suggested that “resting” pancreatic β-cells, by exogenous suppression of blood glucose, can restore function in diabetic states. This is the case for high-fat diet–induced glucose intolerance in Kir6.2^+/−^[AAA] mice [42]. Within 2 wk of restoration of normal diet, Kir6.2^+/−^[AAA] mice recover secretory capacity and glycemic control to near normal [42]. Genetic restoration of excitability has not been attempted in KO mice, but the present study allows us to begin to assess the lability of this effect. First, the pellets are designed to release at the appropriate dose for ∼90 d. The hyperglycemia and hypoinsulinism that develop within a few days of pellet implantation is maintained for at least 6 wk (42 d, Figures 1 and 4), and we did not routinely monitor animals after this time. However, we did assess a small number of animals at 95 and 118 d after implantation, i.e., 5 and 28 d after the pellets should have stopped releasing. By 1 mo, glucose tolerance is restored (Figures 2F and 3F) and even at only 5 d there is marked normalization of blood glucose and glucose tolerance (Figures 2E and 3E). These results strongly demonstrate that insulin secretory failure in response to loss-of-K_ATP_ activity is reversible, if hyperexcitability is removed.

More dramatically, isolated islets from implanted mice reveal rapid reversibility at the cellular level. Within a few hours of removal from the circulating glibenclamide, isolated islets regain not only essentially normal K_ATP_ activity but they regain essentially normal GSIS too. This means that whatever underlying mechanism causes the secretory failure, it is fully and rapidly reversible. Although 5.6 mM glucose is not particularly high, the finding that overnight incubation is sufficient to restore the secretory response to glucose in these islets may have parallels with the recent finding that overnight incubation of SUR1 KO islets in elevated (10 mM) glucose also restores insulin secretory capacity [55].

### Relevance of the Present Findings for Type 2 Diabetes

The present findings have implications both for the potential etiology of type 2 diabetes and for pharmacological intervention. People with type 2 diabetes typically experience a gradual loss of secretory function. In many patients, SUs effectively control glycemia for an extended period [33], but over the long term (months to years) SU therapies often fail [33,56,57]. Several animal studies [28,58,59] and studies of isolated islets and cells [37,52] provide evidence that long-term SU treatment leads to impaired glucose tolerance and GSIS. The present study shows a markedly impaired secretory response to chronic glibenclamide that can be rapidly reversed following removal of the drug in vitro. In the implanted animals, glibenclamide release is continuous, and in patients, dosing is pulsatile. Conceivably, pulsatile presentation may provide islets with unstimulated periods that allow restoration of secretory capacity and responsivity. This rationale may suggest that lower-potency, shorter-acting SUs might be more efficacious therapeutically. In nonobese people with type 2 diabetes, including those in whom SUs secondarily fail, there are reports of significant restoration of β-cell secretory activity after a brief period of intensive insulin therapy [60,61]. This finding may argue for re-evaluation of short-acting analogs and give cause to further consider approaches [62] to reverse loss of secretory response in patients with type 2 diabetes following long-term SU treatment, and thereby prolong the use of pharmacotherapies and delay the need to switch to insulin.

## Abbreviations

GSIS: glucose-stimulated insulin secretion
GTT: glucose tolerance test
HI: hyperinsulinism
ITT: insulin tolerance test
KO: knock-out
SEM: standard error of the mean
SU: sulfonylurea
TUNEL: terminal deoxynucleotidyl transferase biotin-dUTP nick end labeling
WT: wild type
